# Effects of Asprosin and Role of TLR4 as a Biomarker in Endometrial Cancer

**DOI:** 10.3390/molecules30163410

**Published:** 2025-08-18

**Authors:** Rebecca Karkia, Cristina Sisu, Sayeh Saravi, Ioannis Kyrou, Harpal S. Randeva, Jayanta Chatterjee, Emmanouil Karteris

**Affiliations:** 1 College of Health, Medicine and Life Sciences, Brunel University of London, Uxbridge UB8 3PH, UK; rebecca.karkia@nhs.net (R.K.); cristina.sisu@brunel.ac.uk (C.S.); sayeh.saravi@brunel.ac.uk (S.S.); jayanta.chatterjee1@nhs.net (J.C.); 2 Academic Department of Gynaecological Oncology, Royal Surrey NHS Foundation Trust Hospital, Guildford GU2 7XX, UK; 3 Warwickshire Institute for the Study of Diabetes, Endocrinology and Metabolism, University Hospitals Coventry and Warwickshire NHS Trust, Coventry CV2 2DX, UKharpal.randeva@uhcw.nhs.uk (H.S.R.); 4 Aston Medical School, College of Health and Life Sciences, Aston University, Birmingham B4 7ET, UK; 5 Warwick Medical School, University of Warwick, Coventry CV4 7AL, UK; 6 Centre for Sport, Exercise and Life Sciences, Research Institute for Health and Wellbeing, Coventry University, Coventry CV1 5FB, UK; 7 College of Health, Psychology and Social Care, University of Derby, Derby DE22 1GB, UK

**Keywords:** endometrial cancer, asprosin, TLR4, PTPRD, OR4M1, biomarkers

## Abstract

(1) Background: Following the discovery of the adipokine/hormone asprosin, a substantial amount of research has provided evidence for its role in the regulation of glucose homeostasis, as well as appetite, and insulin sensitivity. Its levels are dysregulated in certain disease states, including breast cancer. To date, little is known about its role in endometrial cancer (EC). The present study investigated the effects of asprosin on the transcriptome of the Ishikawa and NOU-1 EC cell lines, and assessed the expression of asprosin’s candidate receptors (TLR4, PTPRD, and OR4M1) in health and disease. (2) Methods: tissue culture, RNA extraction, RNA sequencing, reverse transcription-quantitative PCR, gene enrichment and in silico analyses were used for this study. (3) Results: *TLR4* and *PTPRD* were significantly downregulated in EC when compared to healthy controls. *TLR4* appeared to have a prognostic role in terms of overall survival (OS) in EC patients (i.e., higher expression, better OS). RNA sequencing revealed that asprosin affected 289 differentially expressed genes (DEGs) in Ishikawa cells and 307 DEGs in NOU-1 cells. Pathway enrichment included apoptosis, glycolysis, hypoxia, and PI3K/AKT/ mTOR/NOTCH signalling for Ishikawa-treated cells. In NOU-1, enriched processes included inflammatory response, epithelial-mesenchymal transition, reactive oxygen species pathways, and interferon gamma responses. Other signalling pathways included mTORC1, DNA repair, and p53, amongst others. (4) Conclusions: These findings underscore the importance of understanding receptor dynamics and signalling pathways in the context of asprosin’s role in EC, and provide evidence for a potential role of TLR4 as a diagnostic biomarker.

## 1. Introduction

Endometrial cancer (EC) is the most common gynaecological malignancy in developed countries, with its incidence rising significantly over the last two decades. This increase is largely attributed to an ageing population and escalating obesity rates. Systematic reviews and meta-analyses have identified key risk factors for EC development, including elevated body mass index (BMI), diabetes mellitus, polycystic ovary syndrome (PCOS), and nulliparity [1,2,3]. The histopathologic types of ECs according to WHO [4] are endometrioid carcinoma (i.e., adenocarcinoma; adenocarcinoma-variants), mucinous adenocarcinoma, serous adenocarcinoma, clear cell adenocarcinoma, undifferentiated carcinoma, and neuroendocrine tumours. Approximately 70–80% of ECs are of endometrioid subtype, whilst 10% are serous [5].

In terms of its molecular classification, the European Society of Gynaecological Oncology (ESGO), the European Society for Radiotherapy and Oncology (ESTRO) and the European Society of Medical Oncology (ESMO) sought to stratify patients diagnosed with EC by a different prognostic approach [6]. Prognostic features such as depth of myometrial invasion, lymphovascular space invasion (LVSI), and histological subtype have been incorporated into a risk stratification system, which has subsequently been updated in 2020 with the option of adding molecular data to best reflect differences in prognosis, particularly for those with early-stage disease [7].

Recently, we have shown that the components of metabolic syndrome (MetS), both independently and in combination, significantly increase the risk of EC [8]. Asprosin is a newly discovered adipokine that plays a role in regulating glucose metabolism/insulin sensitivity, and appetite, whilst it is dysregulated in cardiometabolic diseases [8]. For example, higher circulating levels of asprosin were measured in females with obesity than in females with normal BMI, while decreased circulating asprosin levels were noted in females 6 months following bariatric surgery [9]. Similarly, Ugur et al. (2022) studied volunteers of all BMI categories from underweight to class 3 obesity and demonstrated elevated circulating asprosin levels amongst patients with obesity in both serum and saliva [10].

Asprosin’s effects are mediated through different receptors. To date, the primary receptors that have been investigated are the Olfactory Receptor Family 4 Subfamily M Member 1 (OR4M1), Toll Like Receptor 4 (TLR4) and Protein Tyrosine Phosphatase Receptor Delta (PTPRD), though limited evidence for a *bona fide* asprosin receptor exists [11,12,13,14]. Currently, little is known about the effects of asprosin in EC. In this study, we have measured gene and protein expression of asprosin receptors in EC preclinical models and clinical samples, and assessed their prognostic role. We have also investigated the effects of asprosin on the transcriptome of Ishikawa and NOU-1 EC cell lines.

## 2. Results

### 2.1. Expression of Asprosin Receptors in Endometrial Cancer (EC) Cell Lines

**Figure 1:**
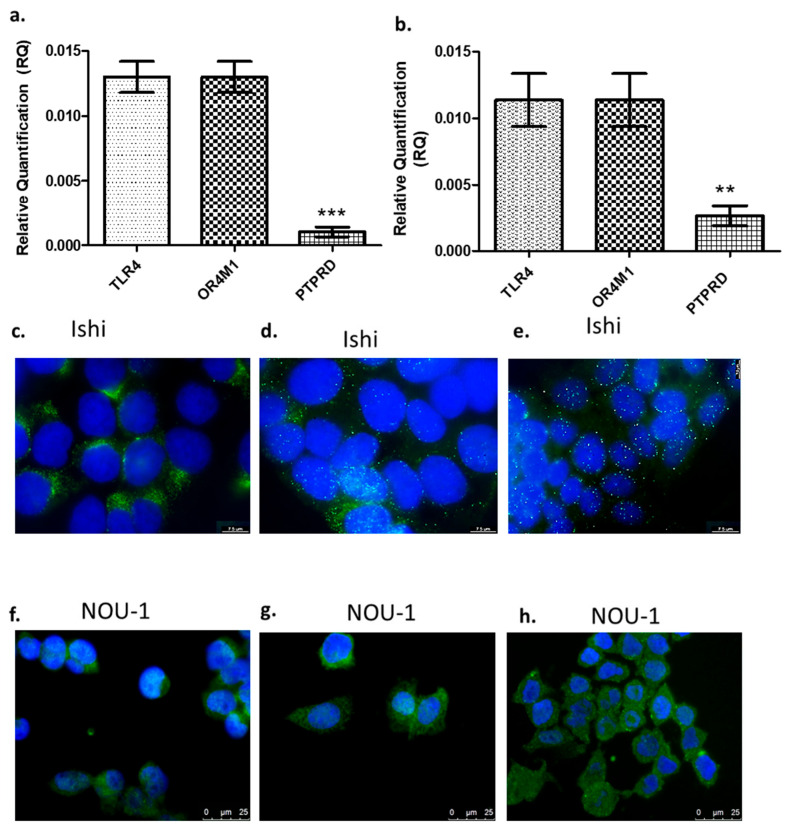
Gene and protein expression of asprosin receptors. Gene expression of *TLR4*, *PTPRD*, and *OR4M1* in Ishikawa (Ishi) cells (**a**) and in NOU-1 cells (**b**). The relative quantification (RQ) was calculated using the delta Ct (ΔΔCt) method. Protein expression of TLR4, PTPRD and OR4M1 in Ishikawa (Ishi; (**c**–**e**)) and NOU-1 cells (**f**–**h**). For immunofluorescence, we have used TLR4 (PA5-23124), OR4M1 (NBP2-46853) and PTPRD (NBP2-49153) primary rabbit antibodies. Anti-rabbit Alexa Fluor 488 was used as a secondary antibody (green), and the DAPI nuclear stain (blue) was used to stain the nuclei. Significance was calculated using one-way ANOVA. *** *p* < 0.0001, ** *p* < 0.001.

In this study, we have used two different EC cell lines, namely Ishikawa and NOU-1. RT-qPCR confirmed the expression of all asprosin’s putative receptors, i.e., *TLR4*, *PTPRD* and *OR4M1* (Figure 1a,b). In both cell lines, *PTPRD*’s expression was significantly lower when compared to *TLR4* or *OR4M1*. Immunofluorescence analysis was also conducted to assess the protein expression of these receptors. A strong cytoplasmic/membrane staining was evident for TLR4 for both cell lines (Figure 1c,f), whereas PTPRD had mainly a scattered cytoplasmic distribution in Ishikawa cells and more membranous in NOU-1 cells (Figure 1d,g). Finally, OR4M1 demonstrated mainly a membrane localisation in Ishikawa cells, in accordance with its role as a G protein-coupled receptor (GPCR; Figure 1e,h).

### 2.2. Differential Expression of TLR4, PTPRD, and OR4M1 in EC: Potential Role of TLR4 as a Biomarker

**Figure 2:**
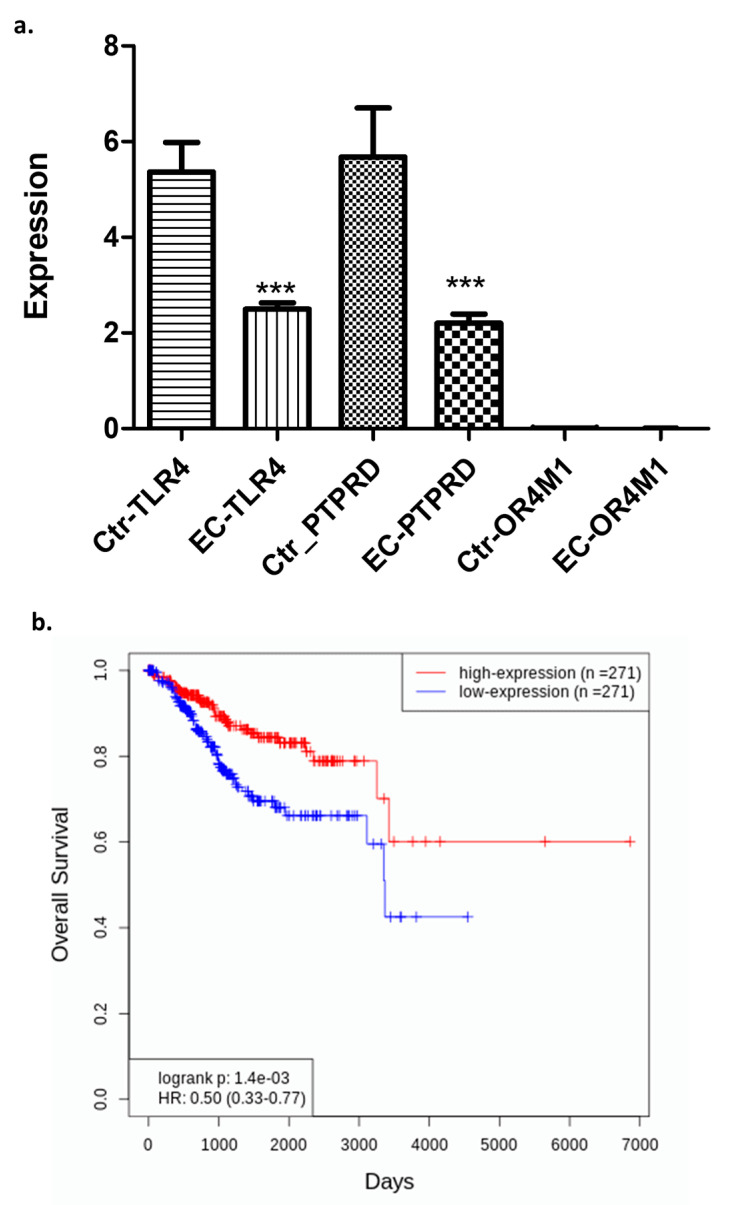
Expression of the *TLR4*, *PTPRD*, and *OR4M1* asprosin receptors in EC patients (*n* = 557) and healthy controls (*n* = 35). (**a**) Downregulation of *TLR4* and *PTPRD*, but not *OR4M1,* in EC patients compared to controls (*** *p* < 0.0001); (**b**) EC patients expressing higher levels of *TLR4* (*n*= 271) have better overall survival compared to EC patients with lower *TLR4* expression (*n* = 271) (*p* = 0.0014).

Curating data from the database OncoDb (TCGA and GTEx), we have assessed the expression of the above-mentioned receptors in EC patients (*n* = 557) and healthy controls (*n* = 35) (Figure 2a). TLR4 was significantly downregulated in EC patients when compared to controls (*p* < 0.0001). Similarly, PTPRD was also significantly downregulated in EC patients when compared to controls (*p* < 0.0001). Very little data were available for OR4M1 to draw any meaningful conclusions, but in general, very low expression was noted in both groups (0.01 to 0.06). Out of all three putative asprosin receptors, only TLR4 appeared to have a prognostic role in terms of overall survival (OS) in EC patients (Figure 2b). Patients with higher expression of TLR4 had higher OS (*n* = 271), when compared to patients with low TLR4 expression (*n* = 271; *p* = 0.0014).

### 2.3. Transcriptomic Changes of Ishikawa and NOU-1 Cells Treated Wth Asprosin

Initially, RNA sequencing analysis was performed on asprosin-treated (10 nM for 4 h) Ishikawa cells and compared to non-treated controls. We have chosen this concentration and time-point based on previous studies from our groups demonstrating that asprosin can induce signalling pathways and transcriptomic changes at these points [13,15].

**Figure 3:**
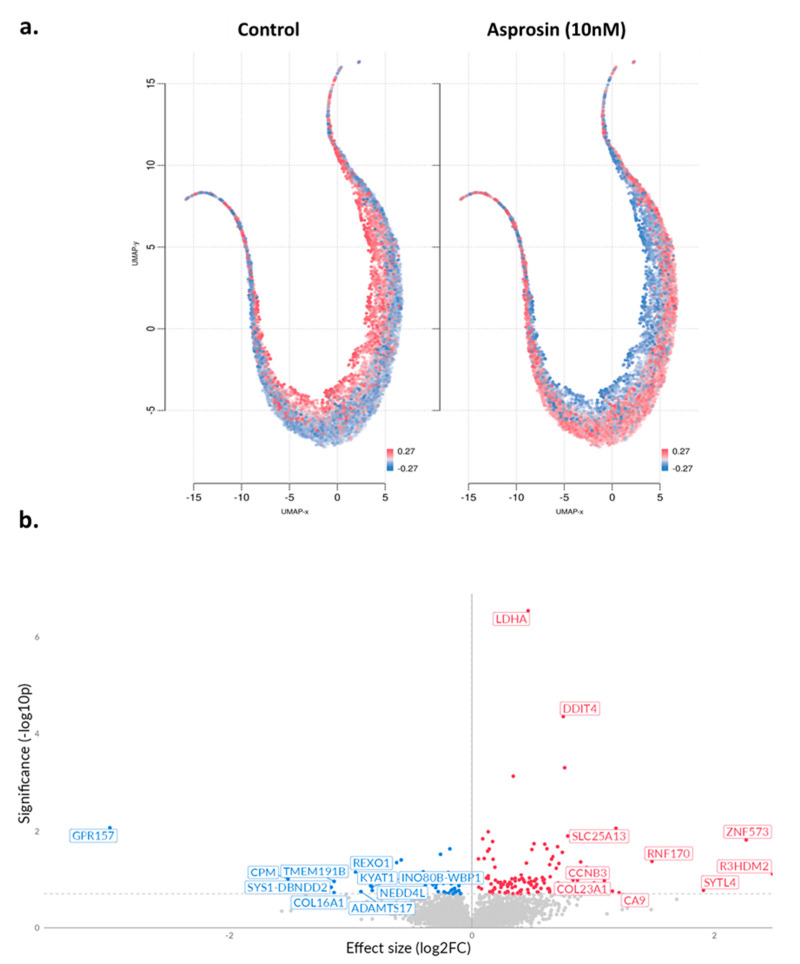
UMAP projections and volcano plot in asprosin-treated Ishikawa cells. (**a**) UMAP projections of Ishikawa cells under two conditions: untreated control and asprosin-treated. Each point corresponds to an individual gene, and red colour indicates higher gene expression, whereas blue indicates lower expression. (**b**) Volcano plot of differentially expressed genes (DEGs) in asprosin-treated Ishikawa cells relative to untreated controls. Upregulated genes with positive log2FC are shown in red, downregulated genes are shown in blue, and non-significant genes are depicted in grey. Selected top hits (e.g., LDHA, DDIT4, GPR157) are labelled. The dashed horizontal line denotes the *p*-value threshold (*p* < 0.05).

A total of 289 differentially expressed genes (DEGs) were identified, of which 186 were up-regulated and 103 down-regulated (Table A1). Figure 3a provides an overview of UMAP clustering coloured by relative log-expression of genes between the different phenotypic groups (−0.27 to 0.27 Z-score of median transcripts per million; TPM). Subsequently, a volcano plot was generated indicating log fold change (log2FC) between treated and untreated cells (Figure 3b).

**Figure 4:**
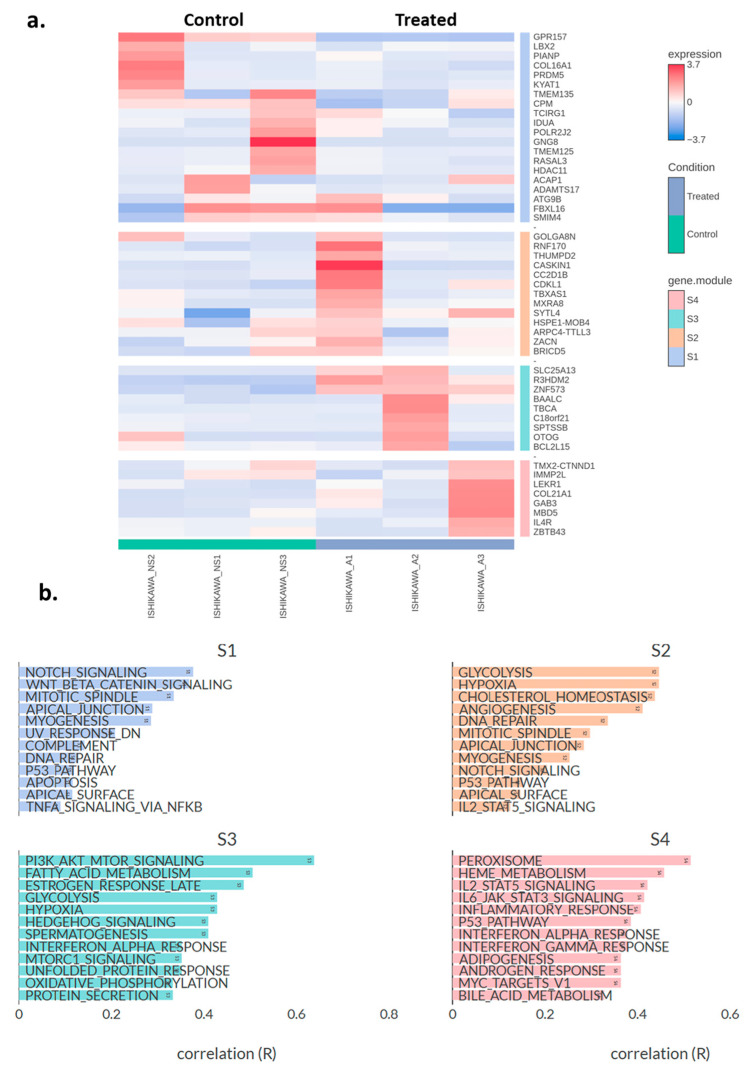
(**a**) Heatmap showing the top 50 differentially expressed genes in asprosin-treated Ishikawa cells (blue-labelled samples) compared to untreated controls (green-labelled samples). The colour gradient indicates standardised expression levels, ranging from low (blue, −2.5) to high (red, +3.7). Hierarchical clustering was performed on both genes and samples, with gene clusters (S1–S4) labelled on the right. A: asprosin-treated, NS: no supplement. (**b**) Pathway correlation analysis for the four gene clusters (S1, S2, S3, and S4). Each bar represents the correlation coefficient (R) between a given hallmark gene set and the module’s gene expression profile. Higher values of R indicate stronger associations between the module and that particular pathway. Ishikawa_A1-A3 and Inshikawa_NS1-3 refer to treated and control samples, respectively (*n* = 3 per group).

Subsequently, hierarchical clustering was performed on the top 50 DEGs (Ishikawa asprosin-treated vs. control (no supplement; NS)), and the genes were grouped into four distinct clusters S1–S4 (Figure 4a). Under S1 cluster, the most prominent functional annotations related to Notch, and Wnt/Beta Catenin signalling, as well as mitotic spindle, apical junction and myogenesis (Figure 4b). In the S2 cluster, main annotations include glycolysis, hypoxia, cholesterol homeostasis and angiogenesis, whereas in S3, the most enriched annotations include PI3K_AKT_mTOR signalling, fatty acid metabolism, and estrogen response. Finally, the S4 cluster included peroxisome, heme metabolism, IL2_STAT5 and IL6_JAK_STAT3 signalling pathways (Figure 4b).

**Figure 5:**
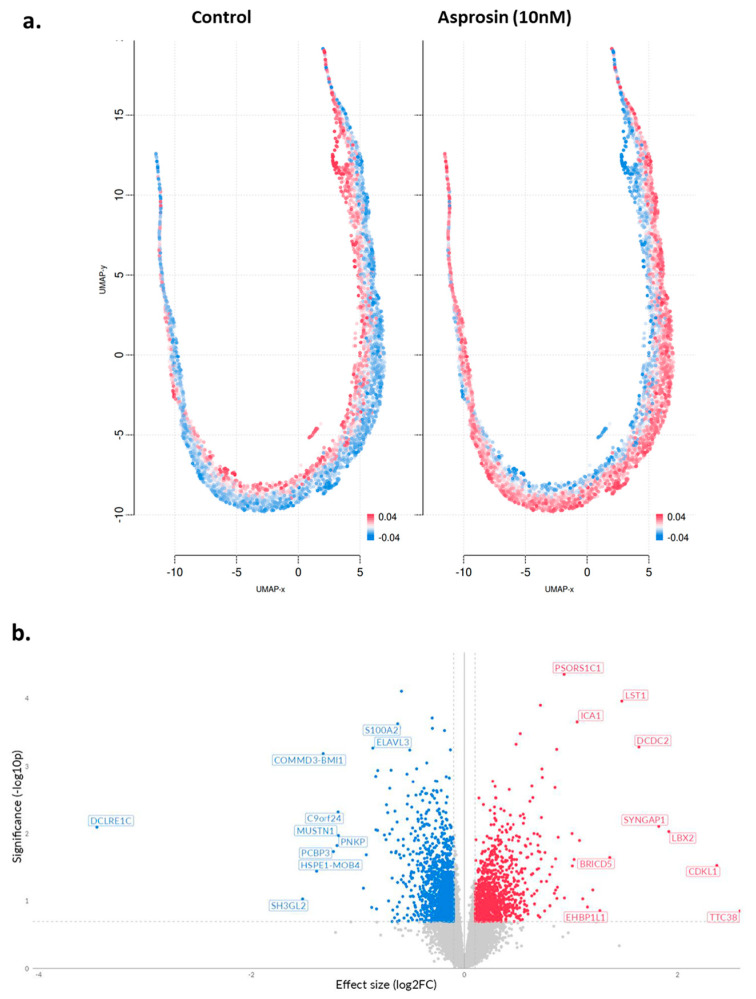
(**a**) UMAP projections of asprosin-treated NOU-1 cells; (**b**) Volcano plot of DEGs in asprosin-treated (10 nM for 4 h) cells when compared to untreated controls. Genes that are significantly upregulated are highlighted in red, whereas significantly downregulated genes are highlighted in blue. Each point represents a single gene; labelled genes (e.g., *COMMD3*, *BMI1*, *DCDC2*, *LST1*, *LBX2*) are among the most significantly altered. The dashed vertical lines mark a 0.1 effect size cutoff, while the dashed horizontal line denotes the *p*-value threshold (*p* < 0.05).

Similar analyses were performed in NOU-1 cells, where 307 DEGs were identified, 202 of which were up-regulated and 105 down-regulated (Table A2). UMAP clustering of genes coloured by relative log-expression of genes between the different groups provided some interesting insights. The treated cells show a distinct shift in expression profiles compared to the control, with clusters of cells exhibiting higher expression (more red regions; Figure 5a). In addition, the volcano plot indicates log2FC changes between asprosin-treated and untreated (control) NOU-1 cells (Figure 5b).

**Figure 6:**
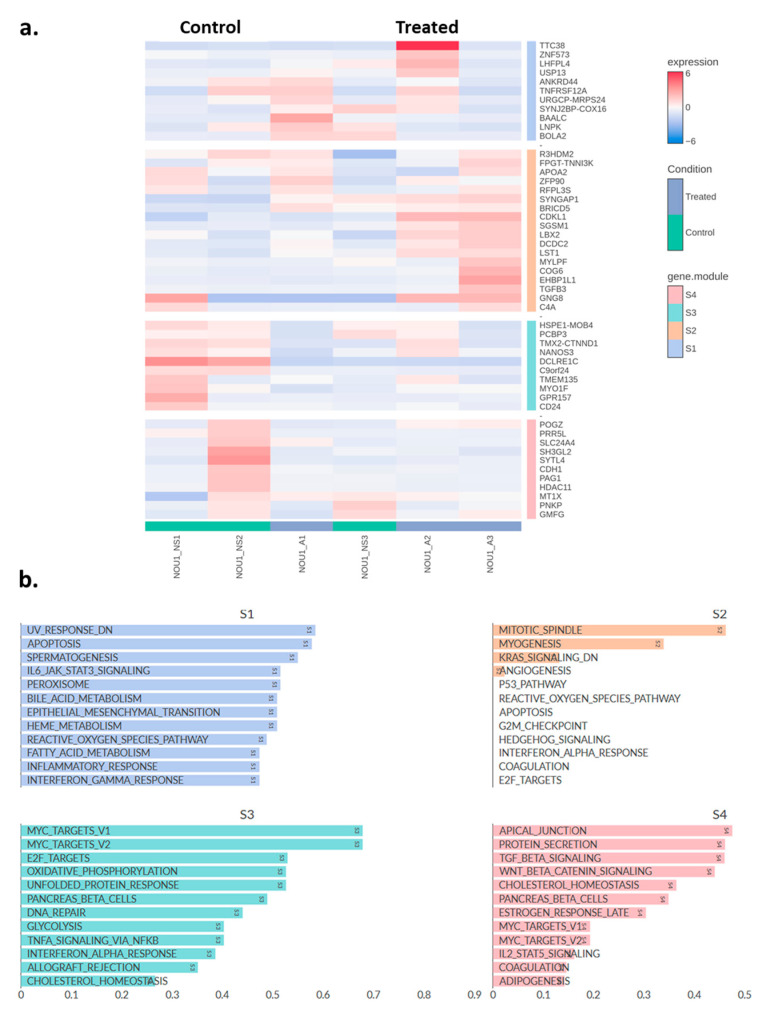
(**a**) Heatmap showing the top 50 differentially expressed genes in asprosin-treated NOU-1 cells (blue-labelled samples) compared to untreated controls (green-labelled samples). Hierarchical clustering was performed on both genes (rows) and samples (columns). A: asprosin-treated, NS: no supplement. (**b**) Pathway correlation analysis for the four gene clusters (S1, S2, S3, and S4) identified in NOU-1 cells treated with asprosin. NOU1_A1-A3 and NOU1_NS1-3 refer to treated and control samples, respectively (*n* = 3 per group).

Using a similar methodology as for the Ishikawa cells, a heatmap of the top 50 DEGs in NOU-1 cells was generated, distributing these genes in four different clusters (Figure 6a). Figure 6b depicts pathway enrichment analysis for four gene clusters (S1–S4), highlighting biological processes associated with asprosin treatment. In S1, pathways such as the inflammatory response, epithelial-mesenchymal transition (EMT), reactive oxygen species (ROS) pathways, and interferon gamma response are enriched. These processes suggest asprosin’s involvement in stress responses, immune signalling, and cellular plasticity, potentially influencing the tumour microenvironment. In S2, key pathways include KRAS signalling, myogenesis and mitotic spindle functioning, indicating that asprosin might affect cell cycle regulation, cancer-related signalling, and vascularisation, all of which are critical for tumour growth and progression. S3 reveals enrichment in MYC target pathways, oxidative phosphorylation, DNA repair, and glycolysis. These pathways are fundamental to cancer metabolism, energy production, and genomic stability, suggesting that asprosin may play a role in metabolic reprogramming and enhancing cellular survival mechanisms. Lastly, S4 is enriched in pathways such as TGF-beta signalling, WNT-beta catenin signalling, protein secretion, and cholesterol homeostasis. These pathways implicate asprosin in intercellular communication, lipid metabolism, and processes associated with cancer stemness and metastasis.

## 3. Discussion

In this study, we provide novel evidence that the human endometrium and two preclinical in vitro models (i.e. Ishikawa and NOU-1 EC cell lines) expresses all three known asprosin receptors (OR4M1, TLR4 and PTPRD), with *TLR4* and *PTPRD* being downregulated in EC. Notably, due to limited data for OR4M1 in EC, it is challenging to draw any conclusions at this stage. We also offer a novel insight into the regulation EC transcriptomes by asprosin in vitro. Although there is not a *bona fide* asprosin receptor, PTPRD has been identified as a potential orexigenic receptor for asprosin in hypothalamic AgRP neurons, since genetic ablation of PTPRD leads to loss of appetite, resistance to diet-induced obesity, and lack of response to asprosin [11]. Similarly, asprosin appears to impair insulin secretion in response to glucose in human primary islets containing β-cells via a TLR4 pathway [12]. We have also shown that asprosin exerts pro-inflammatory effects in THP-1 macrophages in vitro, via activation of TLR4 [13]. Another candidate receptor for asprosin is the olfactory receptor OR4M1, since it can promote gluconeogenesis and maintains glucose homeostasis via this GPCR [14]. Of note, despite the differences on the gene expression of asprosin receptors in our in vitro models, substantial expression was evident when immunofluorescence was performed. Although the protein expression was not quantitative, these discrepancies can arise from the fact that transcription not always is reflected in translation, given the complexity of the regulation steps that need to be followed, especially in cancer [16].

All these three receptors have been shown to be differentially expressed in certain cancers. We have previously shown that PTPRD is dysregulated in patients with glioblastoma and in EC, whilst its expression is significantly downregulated in patients with obesity [17]. In gastric cancers, loss of PTPRD induced CXCL8 and promoted angiogenic and metastatic events, via STAT3 and ERK signalling pathways [17]. PTPRD was also shown to be involved in colon cancer cell migration via a β-catenin/TCF/CD44 signalling pathway, whereas in lung cancer PTPRD appears to act as a tumour suppressor gene [18,19,20]. Notably, PTPRD is mutated in 11.1% of endometrial samples. In a GWAS meta-analysis, 13 loci were associated with EC and endometriosis, with one particular locus located within the PTPRD gene [21]. Collectively, these data point towards a central role of PTPRD not only as a potential tumour suppressor gene, but also as an orexigenic mediator in EC. 

Toll-like receptors (TLRs) are pattern-recognition receptors that detect ligands and initiate downstream signalling involving a number of adaptor molecules (e.g. MyD88) that upon recruitment drives activation of transcription factors (e.g. NF-κB) [22]. For example, TLR4 activation can result in NF-κB translocation to the nucleus, modulating transcription of *COX-2*, a gene related to inflammation [23]. In the same study it was shown that LPS (a ligand for TLR4) induced a TLR4-dependent stimulation of p38, ERK1/2, and JNK. Of note, inhibition of JNK induced NF-κB activity and expression of *COX-2*. Recently, using an in vitro EC model, it was demonstrated that heme metabolism reduces phagocytosis by modulating the secretion of TLR4-mediated IFN Iα as well as CD36 expression; contributing to events leading to immune escape in this cancer [24]. The authors have suggested that an imbalanced immune microenvironment promotes EC progression. Previous studies have also implicated TLR4 in EC. For example, when Ishikawa cells were treated with fusaric acid, it led to a decrease in cell proliferation by compromising the expression of *TLR4* [25]. Of note, a TLR4 polymorphism (rs4986790), did not appear to be associated with EC risk [26].

Here, we show that *TLR4* is downregulated in EC; a finding that corroborates initial studies, where *TLR4* was significantly downregulated in endometrial hyperplasia and adenocarcinoma when compared with controls (i.e. postmenopausal women) [27]. Moreover, we have demonstrated that TLR4 appears to have a potential prognostic role, since EC patients with higher expression have better overall survival. Similarly, TLR4 appears to have a prognostic role in ovarian cancer progression [28]. However, it should be noted that in a meta-analysis study, it was suggested that increased *TLR4* expression is associated with poor OS in patients with solid tumours [29]. Indeed, the finding that high TLR4 expression correlates with better overall survival is intriguing. In a review article by Lupi et al, the complexity of TLR4 signalling in gynecological cancers was discussed at length [30], where the authors suggested that TLR4 can have a dual role exerting both anti- and pro-tumour responses, depending on the pathway it activates. For example, in most cancers, uncontrolled TLR4-mdiatd signalling tips the tumor microenvironment (TME) towards a proliferative status, and evasion of immune surveillance; involving secretion of proinflammatory cytokines and other molecules. On the other hand, activation of TLR4 can enhance an immune response (e.g. T lymphocytes) that will result to inhibition of cancer cell proliferation. Therefore, more research is needed to provide a better insight into the TLR4 pathway(s) in EC and whether its expression can have any clinical utility as a biomarker. However, there is a higher order of complexity when it comes to the ligand binding and TLR4 signalling. Given the multiple ligands that can bind to and activate TLR4, future studies should also elucidate if activation of TLR4 by asprosin can exert anti-proliferative effects and if it acts in TME-specific manner (i.e. well-differentiated vs. poorly-differentiated EC).

Olfactory receptors can detect a wide range of odorants, as well as numerous endogenous ligands. Over the past years, a growing body of studies has pointed towards involvement of olfactory receptors in a number of diseases, including infectious diseases (e.g. COVID-19), neurological diseases, metabolic diseases and cancer [31,32]. Previous work from our group has also shown that *OR4M1* is expressed in the ovaries and is upregulated in early stages of ovarian cancer (I and II) compared to late ones (III and IV) [33]. In the current study, there were limited data available to draw any conclusions regarding the expression or its prognostic value in EC. However, if asprosin influences cAMP signalling through OR4M1, cAMP-Response Element Binding Proteins (CREB) could become activated. CREB binds cAMP-response elements in promoter regions to regulate transcriptional events [34]. A possibility of its low expression, might also have to do with the fact that it belongs to the GPCR family. Indeed, since OR4M1 is a GPCR, it is possible that chronic stimulation by asprosin might lead to desensitization and internalization of this receptor. This reduces the receptor availability on the cell surface and may, in turn, lower mRNA levels which would lead to temporary loss of function. 

In the present study, treatment of two in vitro models has provided a novel insight into the regulation of EC by asprosin. The top biomarkers of asprosin-exposure in Ishikawa cells include: GPR157, ZNF573, R3HDM2, TMEM191B, CA9, SLC24A4, CDK11A, CXCR4. Although there is no data on ZNF573 and EC, it appears to have a diagnostic role for ovarian cancer in combination with other biomarkers [35]. Another upregulated gene is CA9, a hypoxia-responsive gene that is upregulated in the hypoxic tumour microenvironment [36]. Overexpression of CA9 correlates with tumour progression, metastasis, and poor prognosis in cancers including renal carcinomas, cervical squamous carcinomas, oesophageal carcinomas, bladder carcinomas and non-small cell lung carcinomas [37]. Asprosin might drive the increase of *CA9* expression via induction of pro-inflammatory cytokines (e.g., TNF-α, IL-6), which can upregulate hypoxia-response genes, including CA9, even under normoxic conditions. In contrast, asprosin downregulated *GPR157* and *SLC24A4*. Of note GPR157 has been identified as a potential biomarker for endometrioid endometrial carcinoma [38]. In addition, SLC24A4 was downregulated in colon carcinoma cells; it markedly increased their migration potential [39]. It is possible therefore, that asprosin might influence cell invasion/migration of EC cells via downregulation of such genes. There is some evidence that olfactory receptors can indeed internalize, in similar fashion to other GPCRs, involving GPCR specific kinases (GRKs), and β-arrestin [40]. For example, odorants can induce GRK3 translocation to the cell membrane that can mediate olfactory desensitization [41]. In addition, Lefkowitz’s group has shown that when GRK3 was disrupted, the odorant receptor-mediated desensitization was compromised [42]. Finally, Mashukova et al., argued that prolonged exposure of Hana3A cells to odorants, led to β-arrestin 2 accumulation, within intracellular vesicles [43].

It should be noted that, although DDIT-4 was not featured as one of the top differentiating biomarkers of asprosin treatment it was one of the highest overexpressed DEGs. DDIT4 is rapidly induced by various cellular stresses, including hypoxia, heat shock, or endoplasmic reticulum stress. The primary function of DDIT4 has been linked to its role in suppressing the mechanistic target of rapamycin complex 1 (mTORC1), a crucial regulator of cell growth, tumorigenesis, cell aging, and autophagy [44]. Indeed, in ovarian epithelial cells, increased expression of DDIT4 is associated with a decreased level of pro-apoptotic proteins and an elevation in anti-apoptotic protein levels, particularly when RAS oncogene activity is induced. Collectively, these findings suggest that DDIT4 plays a pivotal role in regulating cell survival and growth, particularly under stress conditions [44]. Of note, high *DDIT4* expression correlating with favorable EC prognosis [45]. Contrary, upregulation of DDIT4 can stimulate cell proliferation in gastric epithelial cells [46] and high expression of DDIT4 correlates with more aggressive tumour behaviour and more advanced stages of disease in colorectal cancer patients [44]. Further research is needed to elucidate the relationship between asprosin and DDIT-4 in terms of its involvement in proliferative or apoptotic events in EC. In the present study, the “enriched” genes as biomarkers of asprosin exposure in NOU-1 cells were: DCDC2, BRICD5, PDE11A, PSORS1C1, ACAP1, and PRR11. PRR11 is a gene that plays a significant role in cell cycle progression and is often implicated in cancer progression [47], whilst it is associated with poor prognosis in a number of cancers [48]. For example, when PRR11 was knockdown in ovarian cancer, a decrease in tumour growth was noted [49]. Similarly, suppression of DCDC2 expression led to inhibition of cell proliferation and subsequent metastasis in colon cancer [50]. Table 1 highlights known relevance of top DEGs to EC.

Despite the robustness of the present findings, certain limitations of our study should be acknowledged. We have relied on using in silico data regarding the expression of *TLR4*, *PTPRD* and *OR4M1* in health and disease (EC). Furthermore, we have assessed asprosin’s effects only in vitro, using two EC cell lines, representing different stages of differentiation. Ishikawa cell line was initially established from an endometrial adenocarcinoma from a 39-year-old female. According to ECACC, these cells induced well-differentiated adenocarcinoma in athymic nude mice. This cell line also expresses steroid receptors (estrogen and progesterone). One the other hand, the NOU-1 cell line is a poorly differentiated lethal human endometrial carcinoma cell line, that lacks both estrogen and progesterone receptors [55]. The properties of these cell lines might account for the differences in the transcriptomic landscape we have observed. To further elucidate the role of asprosin in EC and its broader implications in cancer biology, a number of detailed functional studies could be undertaken, particularly examining asprosin’s effects on cell proliferation, apoptosis and migration at different concentrations and time points. we acknowledge that lack of functional studies is a major limitation for this study. These processes are fundamental to cancer progression and metastasis, making them critical targets for understanding asprosin’s role in cancer pathogenesis.

## 4. Materials and Methods

### 4.1. Cell Culture

Endometrial cancer (EC) cells, Ishikawa, and NOU-1 cells were cultured using a complete medium of either Dulbecco’s Modified Eagle’s Medium (DMEM, Gibco, Bleiswijk, The Netherlands), High-Glucose Liquid Medium (Cytiva, Amersham, UK), or RPMI (Cytiva) as per the manufacturers’ instructions and supplemented with 10% Foetal Bovine Serum (FBS, Gibco, Bleiswijk, The Netherlands) and 1% penicillin-streptomycin (Gibco) at 37 °C with 5% CO_2_.

### 4.2. Immunofluorescence (IF)

In preparation for immunofluorescence (IF), an 8 mm coverslip was added to each well of a 6-well plate under a laminar flow cabinet. In the same method used for sub-culture, Ishikawa and NOU-1 cells were resuspended in complete media and incubated for 24 h or until they reached a confluence of approximately 80% on the coverslip. The 6-well plate was then removed from cell culture conditions. Media were aspirated, and cells were washed twice with 1 mL of PBS. Fixation of cells was undertaken using 4% paraformaldehyde (PFA) for five minutes. Repeat washings were undertaken by applying the solution away from the coverslip to avoid the detachment of cells. No permeabilisation of the cell was undertaken. Blocking was then undertaken using 100 μL of 1% bovine serum albumin (BSA) diluted in PBS in each well. Parafilm was used to prevent dehydration, and the slides were left for one hour at room temperature. Next, 100 μL of the primary antibody solution (TLR4, PTPRD, OR4M1 antibodies) diluted in 1% BSA in PBS was applied. Following incubation with the primary antibody, 1 mL of PBS was then used to wash the coverslips three times for five minutes each. The secondary antibody, anti-Rabbit Alexa Fluor 488 antibody (Merck Millipore, Watford, UK), was added to each well at a concentration of 1:200 and left for one hour in the dark at room temperature. A final three washes with PBS were then undertaken. Coverslips were removed from the six-well plate and allowed to air-dry. Glass slides were prepared with 5 μL of mounting medium with DAPI nuclear stain (Vectashield), and cover slides were inverted gently onto the mounting media and left for ten minutes to allow the mounting media to dry. Slides were sealed with clear nail varnish and left to air dry before viewing under a LEICA DM4000 Fluorescent Microscope. All IF analyses were carried out using the LAS-X analysis software (version 3.7.0).

### 4.3. In Silico Tools

The online database OncoDB [56] was accessed to determine expression PTPRD, TLR4 and OR4M1 in UCEC vs. controls, making use of TCGA and GTEx datasets. Enrichment analysis was also performed using Omics playground (v3.44, BigOmics Analytics, Bellinzona, Switzerland) for the function comparison of DEGs in asprosin-treated versus untreated Ishikawa and NOU-1 cells.

### 4.4. RNA Sequencing

RNA was extracted from three technical replicates from two cell lines, Ishikawa and NOU-1 (3 × no supplement control and 3 × asprosin treated), as previously described [17]. Briefly, indexed libraries were submitted to an Illumina NovaSeq (Novogene, Cambridge, UK). Files were compiled using lllumina package bcl2fastq to convert the base call (BCL) binary results to FASTQ. All samples passed internal quality control before sequencing.

The freely available SSH, PuTTY that connects users to the Linux OS was used in the initial steps of data processing. Three programs, Bowtie, TopHat and Cufflinks [57]. As mentioned, using the BigOmics Analytics platform, CSV files were uploaded to the platform to begin analysis. The clustering module performs a holistic clustering analysis of the samples. The main output of this feature is 2-fold: (i) to generate a heatmap of samples and also to provide a plot of samples obtained by principal components analysis (PCA) or t-distributed stochastic embedding algorithms [58,59,60]. The R program ggplot2 was used to better explore and annotate volcano plots.

### 4.5. RT-qPCR

The expression of the genes of interest were quantified on the QuantStudio 7 Flex Real-Time PCR Machine (Applied Biosystems™, Loughborough, UK) using SYBR™ Green PCR Master Mix (Applied Biosystems™, Loughborough, UK). Each qPCR reaction was performed in triplicate on a MicroAmp™ Fast Optical 96-Well Reaction Plate with Barcode, 0.1 mL. Primer sequences were acquired from the Harvard Primer Bank and issued by Sigma Aldrich (Merck, Gillingham, UK) [61] (Table 2).

### 4.6. Statistical Analysis

Differences identified in RT-qPCR experiments were assessed for statistical significance using one-way ANOVA. All statistical tests were performed using GraphPad Prism^®^ software (GraphPad Software, Inc., San Diego, CA, USA). *p*-values < 0.05 were considered significant.

## 5. Conclusions

The present findings underscore the importance of understanding receptor dynamics and signalling pathways in the context of the potential role of asprosin in EC. The outcomes of future functional studies could provide a deeper insight into the biological mechanisms through which asprosin may influence EC, but also identify potential therapeutic targets. For example, if asprosin enhances cell proliferation and migration via TLR4 or PTPRD signalling, inhibiting these receptors could offer a novel strategy for limiting EC progression. Focusing on functional studies (including potential in vivo models) is a critical step in translating such molecular insights on the role of asprosin into actionable therapeutic approaches.

## Figures and Tables

**Table 1:** Role of DEGs in EC.

TOP DEGs	Function in Endometrial Cancer	Reference
DDIT4	High expression correlated to favourable prognosis in progression-free survival and overall survival	[45]
GPR157	Potential biomarker for endometrioid endometrial carcinoma	[38]
CA9	Increased gene expression when compared to controls	[51]
CXCR4	Overexpression associated with enhanced metastatic dissemination	[52]
LDAH	Inhibition results in a reduction of cell growth	[53]
BM1	Silencing suppresses cancer stemness and enhances chemosensitivity	[54]

**Table 2:** List of primers used.

Gene	Forward Primer	Reverse Primer
*YWHAZ*	*AGACGGAAGGTGCTGAGAAA*	*GAAGCATTGGGGATCAAGAA*
*B-ACTIN*	*CAAGATGAGATTGGATGGC*	*CACATTGTGAACTTTGGGG*
*TLR4*	*AGTTGATCTACCAAGCCTTGAGT*	*GCTGGTTGTCCCAAAATCACTTT*
*PTPRD*	*CAGGCGGAAGCGTTAATATCA*	*TTGGCATATCATCTTCAGGTGTC*
*OR4M1*	*TCTGTTAATGTCCTATGCCTTCC*	*AATGTGGGAATAGCAGGTGG*

## Data Availability

The datasets generated and/or analysed during the current study are available upon reasonable request. Researchers interested in accessing the data can contact the corresponding authors. Data on DEGs is provided within Appendix A.

## Abbreviations

The following abbreviations are used in this manuscript:

Endometrial Cancer	EC
body mass index	BMI
polycystic ovary syndrome	PCOS
European Society of Gynaecological Oncology	ESGO
European Society for Radiotherapy and Oncology	ESTRO
European Society of Medical Oncology	ESMO
metabolic syndrome	MetS
Olfactory Receptor Family 4 Subfamily M Member 1	OR4M1
Toll Like Receptor 4	TLR4
Protein Tyrosine Phosphatase Receptor Delta	PTPRD
G protein-coupled receptor	GPCR
transcripts per million	TPM
differentially expressed genes	DEGs
no supplement	NS
epithelial-mesenchymal transition	EMT
reactive oxygen species	ROS
cAMP-Response Element Binding Proteins	CREB
mechanistic target of rapamycin complex 1	mTORC1
paraformaldehyde	PFA
bovine serum albumin	BSA

## Appendix A

**Table A1:** DEGs in asprosin-treated Ishikawa cells.

Gene_ID	Gene (Upregulated)	Gene_ID	Gene (Downregulated)
ENSG00000005961.19	ITGA2B	ENSG00000028277.21	POU2F2
ENSG00000007350.17	TKTL1	ENSG00000079393.21	DUSP13
ENSG00000009950.16	MLXIPL	ENSG00000087253.13	LPCAT2
ENSG00000050767.18	COL23A1	ENSG00000095587.9	TLL2
ENSG00000061656.11	SPAG4	ENSG00000099250.18	NRP1
ENSG00000066336.12	SPI1	ENSG00000100060.18	MFNG
ENSG00000070526.15	ST6GALNAC1	ENSG00000105497.8	ZNF175
ENSG00000074047.22	GLI2	ENSG00000115590.14	IL1R2
ENSG00000074317.11	SNCB	ENSG00000125804.14	FAM182A
ENSG00000074410.14	CA12	ENSG00000132837.15	DMGDH
ENSG00000081051.8	AFP	ENSG00000139318.8	DUSP6
ENSG00000081237.20	PTPRC	ENSG00000144063.4	MALL
ENSG00000100336.18	APOL4	ENSG00000145934.16	TENM2
ENSG00000101441.5	CST4	ENSG00000151376.18	ME3
ENSG00000102287.19	GABRE	ENSG00000151418.12	ATP6V1G3
ENSG00000102878.18	HSF4	ENSG00000159516.9	SPRR2G
ENSG00000103269.14	RHBDL1	ENSG00000162267.12	ITIH3
ENSG00000104419.17	NDRG1	ENSG00000162571.14	TTLL10
ENSG00000104804.8	TULP2	ENSG00000163016.10	ALMS1P1
ENSG00000105650.22	PDE4C	ENSG00000167588.13	GPD1
ENSG00000106631.8	MYL7	ENSG00000169715.15	MT1E
ENSG00000108405.4	P2RX1	ENSG00000175874.10	CREG2
ENSG00000110777.12	POU2AF1	ENSG00000179362.14	HMGN2P46
ENSG00000112715.25	VEGFA	ENSG00000182168.15	UNC5C
ENSG00000114268.12	PFKFB4	ENSG00000183230.18	CTNNA3
ENSG00000114626.18	ABTB1	ENSG00000185271.9	KLHL33
ENSG00000115257.15	PCSK4	ENSG00000185864.17	NPIPB4
ENSG00000115548.17	KDM3A	ENSG00000187243.16	MAGED4B
ENSG00000116014.10	KISS1R	ENSG00000196628.20	TCF4
ENSG00000117586.11	TNFSF4	ENSG00000197714.9	ZNF460
ENSG00000118407.15	FILIP1	ENSG00000204711.9	C9orf135
ENSG00000121966.7	CXCR4	ENSG00000211716.2	TRBV9
ENSG00000122756.15	CNTFR	ENSG00000212724.3	KRTAP2-3
ENSG00000123977.10	DAW1	ENSG00000213309.3	RPL9P18
ENSG00000124116.19	WFDC3	ENSG00000214145.7	LINC00887
ENSG00000124749.17	COL21A1	ENSG00000218208.1	RPS27AP11
ENSG00000126233.2	SLURP1	ENSG00000224194.1	AC008278.3
ENSG00000127129.10	EDN2	ENSG00000227337.1	RPL23AP43
ENSG00000130540.14	SULT4A1	ENSG00000227777.1	RP4-738P11.3
ENSG00000130595.19	TNNT3	ENSG00000228624.7	HDAC2-AS2
ENSG00000132793.12	LPIN3	ENSG00000228793.2	RP1-223B1.1
ENSG00000134107.5	BHLHE40	ENSG00000229660.1	RP5-1142J19.1
ENSG00000134160.15	TRPM1	ENSG00000231704.6	AC004895.4
ENSG00000134365.13	CFHR4	ENSG00000231738.11	TSPAN19
ENSG00000134874.18	DZIP1	ENSG00000231971.6	CT69
ENSG00000137573.14	SULF1	ENSG00000233196.2	GS1-304P7.1
ENSG00000138696.11	BMPR1B	ENSG00000234262.1	RP1-20N18.4
ENSG00000139445.18	FOXN4	ENSG00000234460.2	XXyac-YM21GA2.4
ENSG00000142609.19	CFAP74	ENSG00000236512.1	RP3-336K20__B.2
ENSG00000142973.14	CYP4B1	ENSG00000237664.1	LINC00316
ENSG00000143127.13	ITGA10	ENSG00000240179.1	RPL26P3
ENSG00000147256.12	ARHGAP36	ENSG00000241870.1	CTB-14A14.1
ENSG00000148926.10	ADM	ENSG00000242288.9	BMS1P4-AGAP5
ENSG00000150625.16	GPM6A	ENSG00000242439.1	CTD-2349P21.1
ENSG00000150722.11	PPP1R1C	ENSG00000249464.6	LINC01091
ENSG00000151062.15	CACNA2D4	ENSG00000250337.7	PURPL
ENSG00000151640.13	DPYSL4	ENSG00000250891.2	LINC02208
ENSG00000153237.18	CCDC148	ENSG00000251095.7	RP11-115D19.1
ENSG00000159167.12	STC1	ENSG00000253217.1	KB-1991G8.1
ENSG00000159871.15	LYPD5	ENSG00000254237.6	RP11-115J16.2
ENSG00000160219.12	GAB3	ENSG00000254508.5	FBXO3-DT
ENSG00000162576.17	MXRA8	ENSG00000258599.2	RP11-84C10.1
ENSG00000162931.12	TRIM17	ENSG00000258754.8	LINC01579
ENSG00000163516.14	ANKZF1	ENSG00000259656.1	RP11-325E5.1
ENSG00000163631.17	ALB	ENSG00000260352.1	CTD-2288F12.1
ENSG00000164362.21	TERT	ENSG00000260475.1	RP11-85A1.3
ENSG00000164406.8	LEAP2	ENSG00000260615.1	RPL23AP97
ENSG00000164463.12	CREBRF	ENSG00000260911.2	RP11-196G11.2
ENSG00000164877.19	MICALL2	ENSG00000262213.1	AC144836.1
ENSG00000165061.15	ZMAT4	ENSG00000264655.1	PPIAP54
ENSG00000165495.16	PKNOX2	ENSG00000267123.7	SCAT1
ENSG00000165935.9	SMCO2	ENSG00000267674.1	RP11-813F20.2
ENSG00000167178.16	ISLR2	ENSG00000267737.1	AC061992.2
ENSG00000167612.13	ANKRD33	ENSG00000268266.1	AC003005.2
ENSG00000167702.13	KIFC2	ENSG00000268350.8	FAM156A
ENSG00000168060.16	NAALADL1	ENSG00000269489.1	RP11-98D18.17
ENSG00000168209.6	DDIT4	ENSG00000270049.3	ATP6V0D1-DT
ENSG00000170074.19	FAM153A	ENSG00000271021.1	RP5-878I13.2
ENSG00000170989.10	S1PR1	ENSG00000271185.1	RP5-855F16.1
ENSG00000171388.12	APLN	ENSG00000271200.1	PATJ-DT
ENSG00000172238.6	ATOH1	ENSG00000271369.1	RP11-350D17.3
ENSG00000172650.15	AGAP5	ENSG00000271803.1	RP1-63M2.5
ENSG00000175018.13	TEX36	ENSG00000272646.1	RP11-188P17.2
ENSG00000175265.18	GOLGA8A	ENSG00000272989.1	LINC02012
ENSG00000175766.13	EIF4E1B	ENSG00000273143.2	DUSP5-DT
ENSG00000176171.11	BNIP3	ENSG00000273321.1	RP11-621L6.3
ENSG00000177453.7	NIM1K	ENSG00000273384.1	RP5-1098D14.1
ENSG00000178015.5	GPR150	ENSG00000273542.2	H4C12
ENSG00000181856.15	SLC2A4	ENSG00000274012.1	RN7SL2
ENSG00000182310.15	SPACA6	ENSG00000274549.1	RP11-676B18.2
ENSG00000182511.12	FES	ENSG00000274813.1	RP11-21I4.3
ENSG00000182985.18	CADM1	ENSG00000274818.1	RP1-292L20.3
ENSG00000184144.12	CNTN2	ENSG00000275409.1	RP11-131L12.4
ENSG00000184564.11	SLITRK6	ENSG00000276077.4	CH507-254M2.2
ENSG00000184786.6	DYNLT2	ENSG00000277027.1	RMRP
ENSG00000184925.12	LCN12	ENSG00000277067.4	CH507-254M2.1
ENSG00000185101.13	ANO9	ENSG00000277991.4	CH507-338C24.1
ENSG00000185189.18	NRBP2	ENSG00000278558.5	TMEM191B
ENSG00000187492.9	CDHR4	ENSG00000282787.1	RP11-151A10.3
ENSG00000197358.9	BNIP3P1	ENSG00000284696.2	LINC02808
ENSG00000197745.3	SCGB1D4	ENSG00000284735.1	RP4-736L20.4
ENSG00000197837.3	H4-16	ENSG00000285201.1	RP4-552O12.3
ENSG00000203685.10	STUM	ENSG00000286064.1	RP4-657E11.11
ENSG00000203780.12	FANK1		
ENSG00000204420.10	MPIG6B		
ENSG00000205702.11	CYP2D7		
ENSG00000214140.11	PRCD		
ENSG00000215252.12	GOLGA8B		
ENSG00000223718.3	AC093107.7		
ENSG00000223756.7	TSSC2		
ENSG00000224635.2	RP4-564F22.5		
ENSG00000225255.6	PSLNR		
ENSG00000225605.3	RP11-550H2.1		
ENSG00000226043.2	AP000705.7		
ENSG00000226250.2	LINC00408		
ENSG00000226321.5	CROCC2		
ENSG00000226956.1	AP000432.2		
ENSG00000227877.7	MRLN		
ENSG00000228224.3	NACA4P		
ENSG00000228232.1	GAPDHP1		
ENSG00000229474.8	PATL2		
ENSG00000229609.1	LINC01079		
ENSG00000230216.1	HSPB1P2		
ENSG00000230323.6	LINC00159		
ENSG00000231359.3	AC072052.7		
ENSG00000231650.1	RFESDP1		
ENSG00000231720.1	RP11-568A7.3		
ENSG00000232065.1	LINC01063		
ENSG00000232354.8	VIPR1-AS1		
ENSG00000233090.1	AC015922.7		
ENSG00000233198.4	RNF224		
ENSG00000233928.6	RP11-305F18.1		
ENSG00000234648.1	AL162151.3		
ENSG00000235308.1	RP11-82K18.2		
ENSG00000236581.9	STARD13-AS		
ENSG00000241529.3	RN7SL767P		
ENSG00000242101.3	RN7SL416P		
ENSG00000242338.6	BMS1P4		
ENSG00000244573.3	RPL30P11		
ENSG00000245750.10	DRAIC		
ENSG00000245870.4	LINC00682		
ENSG00000247095.3	MIR210HG		
ENSG00000248099.4	INSL3		
ENSG00000248459.1	RP11-93K22.1		
ENSG00000249700.9	SRD5A3-AS1		
ENSG00000249937.8	LINC02223		
ENSG00000249965.2	CDC42P4		
ENSG00000250122.1	CTD-2013M15.1		
ENSG00000250241.6	RP11-9G1.3		
ENSG00000250305.9	TRMT9B		
ENSG00000254599.1	RP11-115E19.1		
ENSG00000254701.3	RP11-1415C14.4		
ENSG00000254842.6	LINC02551		
ENSG00000255647.3	CTD-2373J6.2		
ENSG00000255966.1	RP5-940J5.3		
ENSG00000256995.8	RP11-114G22.1		
ENSG00000259039.3	RP11-409I10.2		
ENSG00000259315.1	ACTG1P17		
ENSG00000259704.2	CTD-3094K11.1		
ENSG00000260025.1	CRIM1-DT		
ENSG00000261302.6	NUP93-DT		
ENSG00000261341.7	CTD-2568A17.1		
ENSG00000261433.1	CTC-297N7.1		
ENSG00000261786.1	RP4-555D20.2		
ENSG00000262117.5	BCAR4		
ENSG00000263154.1	RP11-1055B8.2		
ENSG00000265542.5	RP11-60A24.3		
ENSG00000265690.7	RP11-5A19.5		
ENSG00000266714.9	MYO15B		
ENSG00000270069.1	MIR222HG		
ENSG00000270917.1	RP11-27I1.6		
ENSG00000272128.1	KB-1836B5.4		
ENSG00000272645.3	GTF2IP20		
ENSG00000272784.1	RP11-335L23.5		
ENSG00000272795.1	GAR1-DT		
ENSG00000273437.1	RP11-434H6.7		
ENSG00000273712.1	RP5-874C20.7		
ENSG00000274156.1	RP11-407N8.6		
ENSG00000277453.1	CTC-492K19.7		
ENSG00000278595.1	RP5-1068H6.6		
ENSG00000279208.1	bP-21264C1.1		
ENSG00000284523.2	RP13-968A2.1		
ENSG00000285287.1	RP11-643E14.2		
ENSG00000286794.1	GHc-351F8.3		
ENSG00000287542.1	RP11-2I7.1		
ENSG00000287737.1	RP13-516M14.11		

**Table A2:** DEGs in asprosin-treated NOU-1 cells.

Gene_ID	Gene (Upregulated)	Gene_ID	Gene (Downregulated)
ENSG00000170419.11	VSTM2A	ENSG00000058335.16	RASGRF1
ENSG00000008517.19	IL32	ENSG00000135362.14	PRR5L
ENSG00000102057.10	KCND1	ENSG00000158560.14	DYNC1I1
ENSG00000109339.24	MAPK10	ENSG00000189292.16	ALKAL2
ENSG00000132837.15	DMGDH	ENSG00000244128.7	LINC01322
ENSG00000140853.16	NLRC5	ENSG00000287193.1	RP13-20L14.11
ENSG00000155511.18	GRIA1	ENSG00000105976.16	MET
ENSG00000184908.19	CLCNKB	ENSG00000125207.7	PIWIL1
ENSG00000215478.8	CES5AP1	ENSG00000148346.12	LCN2
ENSG00000229140.11	CCDC26	ENSG00000152670.19	DDX4
ENSG00000234722.5	LINC01287	ENSG00000163993.7	S100P
ENSG00000271138.1	IGLVIVOR22-1	ENSG00000165495.16	PKNOX2
ENSG00000272255.1	CTD-3224K15.3	ENSG00000169862.19	CTNND2
ENSG00000274012.1	RN7SL2	ENSG00000173083.16	HPSE
ENSG00000003147.19	ICA1	ENSG00000179520.11	SLC17A8
ENSG00000077092.19	RARB	ENSG00000189129.14	PLAC9
ENSG00000100884.9	CPNE6	ENSG00000204140.10	CLPSL1
ENSG00000106128.19	GHRHR	ENSG00000206172.8	HBA1
ENSG00000120555.13	SEPTIN7P9	ENSG00000224535.2	LINC02771
ENSG00000120907.18	ADRA1A	ENSG00000230216.1	HSPB1P2
ENSG00000124440.16	HIF3A	ENSG00000232732.11	AC073043.1
ENSG00000130377.14	ACSBG2	ENSG00000237126.8	AC073254.1
ENSG00000130592.17	LSP1	ENSG00000239705.2	RP11-65N13.8
ENSG00000147443.13	DOK2	ENSG00000248538.8	RP11-115J16.1
ENSG00000156113.24	KCNMA1	ENSG00000253816.3	GUSBP14
ENSG00000158482.10	SNX29P1	ENSG00000261314.1	RP11-359E8.5
ENSG00000169064.13	ZBBX	ENSG00000261341.7	CTD-2568A17.1
ENSG00000169877.10	AHSP	ENSG00000266950.1	CTD-2623N2.5
ENSG00000170374.6	SP7	ENSG00000271734.1	RP1-111B22.3
ENSG00000175265.18	GOLGA8A	ENSG00000272361.2	GS1-166A23.2
ENSG00000179954.16	SSC5D	ENSG00000273375.1	RP11-803P9.1
ENSG00000182621.18	PLCB1	ENSG00000276832.2	CDRT15P6
ENSG00000186642.16	PDE2A	ENSG00000277764.1	ENSG10010137820.1
ENSG00000188257.12	PLA2G2A	ENSG00000286102.1	FAM246A
ENSG00000188517.16	COL25A1	ENSG00000006606.9	CCL26
ENSG00000189068.11	VSTM1	ENSG00000018236.15	CNTN1
ENSG00000189149.12	CRYM-AS1	ENSG00000107105.15	ELAVL2
ENSG00000214193.11	SH3D21	ENSG00000107954.10	NEURL1
ENSG00000223855.2	HRAT92	ENSG00000110203.9	FOLR3
ENSG00000224389.9	C4B	ENSG00000111319.13	SCNN1A
ENSG00000224637.1	PDSS1P2	ENSG00000116016.14	EPAS1
ENSG00000225783.8	MIAT	ENSG00000124507.11	PACSIN1
ENSG00000226889.3	RP11-474I16.8	ENSG00000124788.19	ATXN1
ENSG00000227438.1	AP001471.1	ENSG00000131620.17	ANO1
ENSG00000228526.7	MIR34AHG	ENSG00000134121.10	CHL1
ENSG00000230096.1	RP11-34C15.2	ENSG00000134531.10	EMP1
ENSG00000232801.1	SDCBPP3	ENSG00000140682.19	TGFB1I1
ENSG00000242866.10	STRC	ENSG00000141668.10	CBLN2
ENSG00000245532.9	NEAT1	ENSG00000144668.12	ITGA9
ENSG00000247982.6	LINC00926	ENSG00000151490.15	PTPRO
ENSG00000249476.2	CTD-2587M2.1	ENSG00000158517.15	NCF1
ENSG00000253848.1	RP11-10N23.5	ENSG00000164398.15	ACSL6
ENSG00000254833.1	RP11-50B3.2	ENSG00000172349.18	IL16
ENSG00000256091.1	MTRF1LP1	ENSG00000173926.6	MARCHF3
ENSG00000260628.5	RP11-1166P10.1	ENSG00000175564.13	UCP3
ENSG00000262877.5	RP11-1055B8.4	ENSG00000176358.15	TAC4
ENSG00000263946.1	RP11-434D2.11	ENSG00000182676.5	PPP1R27
ENSG00000267505.1	CTC-296K1.3	ENSG00000185078.3	RPL9P14
ENSG00000269993.1	AF003625.3	ENSG00000185847.8	LINC01405
ENSG00000271265.1	RP11-230C9.4	ENSG00000198838.14	RYR3
ENSG00000272192.1	PDE7A-DT	ENSG00000204625.10	HCG9
ENSG00000280325.1	AC074183.3	ENSG00000205277.10	MUC12
ENSG00000286379.1	RP11-268J15.6	ENSG00000214243.3	AC004980.10
ENSG00000287263.1	CTD-2201E18.8	ENSG00000224382.2	LINC00703
ENSG00000287331.1	RP11-29H20.2	ENSG00000224566.2	FAM96AP2
ENSG00000011590.14	ZBTB32	ENSG00000225099.1	ATP6V1E1P1
ENSG00000055732.13	MCOLN3	ENSG00000226856.7	THORLNC
ENSG00000056558.11	TRAF1	ENSG00000228232.1	GAPDHP1
ENSG00000066032.19	CTNNA2	ENSG00000228541.1	AC093159.1
ENSG00000066405.13	CLDN18	ENSG00000229203.1	AC103564.7
ENSG00000072422.17	RHOBTB1	ENSG00000229914.1	RP11-404O13.4
ENSG00000073605.19	GSDMB	ENSG00000230268.3	SSU72P8
ENSG00000090238.12	YPEL3	ENSG00000231327.1	LINC01816
ENSG00000099338.23	CATSPERG	ENSG00000232028.1	AC007391.2
ENSG00000102271.14	KLHL4	ENSG00000232065.1	LINC01063
ENSG00000105339.11	DENND3	ENSG00000232599.1	RP1-161N10.1
ENSG00000108602.18	ALDH3A1	ENSG00000233017.3	RP5-908M14.5
ENSG00000108849.8	PPY	ENSG00000233084.2	RPL23AP25
ENSG00000111644.8	ACRBP	ENSG00000234175.1	RP11-730A19.9
ENSG00000120645.12	IQSEC3	ENSG00000234318.1	RP4-771M4.3
ENSG00000126583.12	PRKCG	ENSG00000235126.1	AC128709.3
ENSG00000128655.18	PDE11A	ENSG00000235214.1	FAM83C-AS1
ENSG00000130518.17	IQCN	ENSG00000235828.5	RPL36AP26
ENSG00000130653.16	PNPLA7	ENSG00000243069.8	ARHGEF26-AS1
ENSG00000131480.9	AOC2	ENSG00000249148.2	AC006445.7
ENSG00000132205.11	EMILIN2	ENSG00000250662.1	HNRNPKP5
ENSG00000132259.13	CNGA4	ENSG00000254731.1	CTD-2005H7.1
ENSG00000137098.14	SPAG8	ENSG00000255362.1	LINC02761
ENSG00000138795.10	LEF1	ENSG00000256312.1	RP13-977J11.8
ENSG00000139890.10	REM2	ENSG00000258754.8	LINC01579
ENSG00000144868.14	TMEM108	ENSG00000261564.1	RP11-616M22.10
ENSG00000146038.12	DCDC2	ENSG00000263711.6	LINC02864
ENSG00000151790.9	TDO2	ENSG00000271002.1	RP11-599B13.8
ENSG00000153071.15	DAB2	ENSG00000272057.1	ANKH-DT
ENSG00000154529.15	CNTNAP3B	ENSG00000275773.1	RP11-343D24.2
ENSG00000159958.7	TNFRSF13C	ENSG00000277602.1	AC005363.11
ENSG00000160201.12	U2AF1	ENSG00000278077.1	RP11-157L3.10
ENSG00000161509.14	GRIN2C	ENSG00000278276.1	RP3-324O17.8
ENSG00000162777.17	DENND2D	ENSG00000278463.2	H2AC4
ENSG00000163017.14	ACTG2	ENSG00000280331.1	RP11-545D22.1
ENSG00000163083.6	INHBB	ENSG00000283698.1	VINAC1P
ENSG00000164037.17	SLC9B1	ENSG00000286064.1	RP4-657E11.11
ENSG00000164330.17	EBF1	ENSG00000286508.1	AC000050.1
ENSG00000166833.22	NAV2	ENSG00000286850.1	RP11-533K9.5
ENSG00000167037.19	SGSM1	ENSG00000287961.1	CTD-2562J15.8
ENSG00000167588.13	GPD1		
ENSG00000168785.8	TSPAN5		
ENSG00000169885.10	CALML6		
ENSG00000172005.11	MAL		
ENSG00000172014.12	ANKRD20A4P		
ENSG00000172232.10	AZU1		
ENSG00000174469.23	CNTNAP2		
ENSG00000175097.8	RAG2		
ENSG00000180712.4	LINC02363		
ENSG00000183929.7	DUSP5P1		
ENSG00000185215.11	TNFAIP2		
ENSG00000186115.13	CYP4F2		
ENSG00000187045.19	TMPRSS6		
ENSG00000188710.3	QRFP		
ENSG00000188981.11	MSANTD1		
ENSG00000196338.14	NLGN3		
ENSG00000196421.9	C20orf204		
ENSG00000196604.13	POTEF		
ENSG00000197558.13	SSPOP		
ENSG00000197714.9	ZNF460		
ENSG00000197826.12	CFAP299		
ENSG00000197971.16	MBP		
ENSG00000204540.11	PSORS1C1		
ENSG00000213199.8	ASIC3		
ENSG00000214279.13	SCART1		
ENSG00000214402.7	LCNL1		
ENSG00000214784.4	RPS3AP21		
ENSG00000214900.11	LINC01588		
ENSG00000218208.1	RPS27AP11		
ENSG00000220161.4	LINC02076		
ENSG00000223749.11	MIR503HG		
ENSG00000224019.1	RPL21P32		
ENSG00000224473.1	CCND3P1		
ENSG00000224934.5	RP11-441O15.3		
ENSG00000225156.2	AC012354.6		
ENSG00000225828.1	FAM229A		
ENSG00000226229.2	RPLP0P1		
ENSG00000226314.8	ZNF192P1		
ENSG00000226985.7	LINC01203		
ENSG00000227036.8	LINC00511		
ENSG00000227232.5	WASH7P		
ENSG00000228649.9	SNHG26		
ENSG00000229807.13	XIST		
ENSG00000229869.1	RP11-363N22.2		
ENSG00000230914.3	KIF19BP		
ENSG00000230994.1	FGFR3P1		
ENSG00000231057.4	RP11-122M14.1		
ENSG00000231120.3	BTF3P10		
ENSG00000233178.7	RP11-88I18.2		
ENSG00000234296.1	RP13-16H11.7		
ENSG00000234367.1	PFN1P3		
ENSG00000234498.3	RPL13AP20		
ENSG00000237094.12	RP4-669L17.4		
ENSG00000242715.8	CCDC169		
ENSG00000243802.2	RP11-390K5.1		
ENSG00000245017.2	LINC02453		
ENSG00000245750.10	DRAIC		
ENSG00000246922.9	UBAP1L		
ENSG00000248124.8	RRN3P1		
ENSG00000250420.8	AACSP1		
ENSG00000251221.1	LINC01337		
ENSG00000253641.6	LINCR-0001		
ENSG00000254632.2	RP11-21L23.4		
ENSG00000255452.1	RP11-107P7.1		
ENSG00000255741.1	RP11-757G1.5		
ENSG00000256732.1	RP11-407A16.3		
ENSG00000259030.8	FPGT-TNNI3K		
ENSG00000259156.7	CHEK2P2		
ENSG00000259215.1	RP11-253M7.4		
ENSG00000260236.1	RP11-708J19.1		
ENSG00000260911.2	RP11-196G11.2		
ENSG00000261408.7	TEN1-CDK3		
ENSG00000266274.2	RN7SL138P		
ENSG00000267224.1	AC005498.4		
ENSG00000268307.1	LINC02560		
ENSG00000270019.1	RP11-141B14.1		
ENSG00000270248.1	CTC-471J1.10		
ENSG00000271327.1	RP11-1109F11.3		
ENSG00000272631.1	RP11-359E3.4		
ENSG00000272645.3	GTF2IP20		
ENSG00000272662.1	TIMMDC1-DT		
ENSG00000272942.1	CTA-246H3.12		
ENSG00000273230.1	RP11-1246C19.1		
ENSG00000275265.1	RP11-15J22.8		
ENSG00000277112.3	ANKRD20A21P		
ENSG00000277157.2	H4C4		
ENSG00000278546.1	RP1-41C23.4		
ENSG00000278683.1	RP11-132A1.6		
ENSG00000279161.1	CTB-12A17.2		
ENSG00000279617.1	AC005796.2		
ENSG00000280047.1	CTC-463A16.1		
ENSG00000283189.2	RP11-949J7.8		
ENSG00000286058.1	RP11-32C20.1		
ENSG00000286562.1	RP1-128O3.7		
ENSG00000286656.1	CTD-2201E18.7		
ENSG00000287506.1	RP11-443G13.4		
ENSG00000287766.1	AC006130.5		

## References

[1] SHutt D., Mihaies E., Karteris A., Michael A., Payne M., Chatterjee J. (2021). Statistical Meta-Analysis of Risk Factors for Endometrial Cancer and Development of a Risk Prediction Model Using an Artificial Neural Network Algorithm. Cancers.

[2] Johnson J.-E., Daley D., Tarta C., Stanciu P.I. (2023). Risk of endometrial cancer in patients with polycystic ovarian syndrome: A meta-analysis. Oncol. Lett..

[3] Zhang Y., Liu H., Yang S., Zhang J., Qian L., Chen X. (2014). Overweight, Obesity and Endometrial Cancer Risk: Results from a Systematic Review and Meta-Analysis. Int. J. Biol. Markers.

[4] Höhn A.K., Brambs C.E., Hiller G.G.R., May D., Schmoeckel E., Horn L.C. (2021). 2020 WHO Classification of Female Genital Tumors. Geburtshilfe Frauenheilkd..

[5] Murali R., Soslow R.A., Weigelt B. (2014). Classification of endometrial carcinoma: More than two types. Lancet Oncol..

[6] Colombo N., Creutzberg C., Amant F., Bosse T., González-Martín A., Ledermann J., Marth C., Nout R., Querleu D., Mirza M.R. (2016). ESMO-ESGO-ESTRO Consensus Conference on Endometrial Cancer: Diagnosis, treatment and follow-up. Ann. Oncol..

[7] Concin N., Matias-Guiu X., Vergote I., Cibula D., Mirza M.R., Marnitz S., Ledermann J., Bosse T., Chargari C., Fagotti A. (2021). ESGO/ESTRO/ESP guidelines for the management of patients with endometrial carcinoma. Int. J. Gynecol. Cancer.

[8] Karkia R., Maccarthy G., Payne A., Karteris E., Pazoki R., Chatterjee J. (2025). The Association Between Metabolic Syndrome and the Risk of Endometrial Cancer in Pre- and Post-Menopausal Women: A UK Biobank Study. J. Clin. Med..

[9] Wang C.-Y., Lin T.-A., Liu K.-H., Liao C.-H., Liu Y.-Y., Wu V.C.-C., Wen M.-S., Yeh T.-S. (2019). Serum asprosin levels and bariatric surgery outcomes in obese adults. Int. J. Obes..

[10] Ugur K., Erman F., Turkoglu S., Aydin Y., Aksoy A., Lale A., Karagöz Z.K., Ugur I., Akkoc R.F., Yalniz M. (2022). Asprosin, visfatin and subfatin as new biomarkers of obesity and metabolic syndrome. Eur. Rev. Med. Pharmacol.Sci..

[11] Mishra I., Xie W.R., Bournat J.C., He Y., Wang C., Silva E.S., Liu H., Ku Z., Chen Y., Erokwu B.O. (2022). Protein tyrosine phosphatase receptor δ serves as the orexigenic asprosin receptor. Cell Metab..

[12] Lee T., Yun S., Jeong J.H., Jung T.W. (2019). Asprosin impairs insulin secretion in response to glucose and viability through TLR4/JNK-mediated inflammation. Mol. Cell. Endocrinol..

[13] Shabir K., Gharanei S., Orton S., Patel V., Chauhan P., Karteris E., Randeva H.S., Brown J.E., Kyrou I. (2022). Asprosin Exerts Pro-Inflammatory Effects in THP-1 Macrophages Mediated via the Toll-like Receptor 4 (TLR4) Pathway. Int. J. Mol. Sci..

[14] Li E., Shan H., Chen L., Long A., Zhang Y., Liu Y., Jia L., Wei F., Han J., Li T. (2019). OLFR734 Mediates Glucose Metabolism as a Receptor of Asprosin. Cell Metab..

[15] Kerslake R., Sisu C., Panfilov S., Hall M., Khan N., Jeyaneethi J., Randeva H., Kyrou I., Karteris E. (2022). Differential Regulation of Genes by the Glucogenic Hormone Asprosin in Ovarian Cancer. J. Clin. Med..

[16] Maggi L.B., Weber J.D. (2013). Forget Transcription: Translation Is Where the Action Is. Mol. Cell. Biol..

[17] Orton S., Karkia R., Mustafov D., Gharanei S., Braoudaki M., Filipe A., Panfilov S., Saravi S., Khan N., Kyrou I. (2024). In Silico and In Vitro Mapping of Receptor-Type Protein Tyrosine Phosphatase Receptor Type D in Health and Disease: Implications for Asprosin Signalling in Endometrial Cancer and Neuroblastoma. Cancers.

[18] Bae W.J., Ahn J.M., Byeon H.E., Kim S., Lee D. (2019). PTPRD-inactivation-induced CXCL8 promotes angiogenesis and metastasis in gastric cancer and is inhibited by metformin. J. Exp. Clin. Cancer Res..

[19] Kohno T., Otsuka A., Girard L., Sato M., Iwakawa R., Ogiwara H., Sanchez-Cespedes M., Minna J.D., Yokota J. (2010). A catalog of genes homozygously deleted in human lung cancer and the candidacy of *PTPRD* as a tumor suppressor gene. Genes Chromosomes Cancer.

[20] Funato K., Yamazumi Y., Oda T., Akiyama T. (2011). Tyrosine phosphatase PTPRD suppresses colon cancer cell migration in coordination with CD44′. Exp. Ther. Med..

[21] Painter J.N., O’mara T.A., Morris A.P., Cheng T.H., Gorman M., Martin L., Hodson S., Jones A., Martin N.G., Gordon S. (2018). Genetic overlap between endometriosis and endometrial cancer: Evidence from cross-disease genetic correlation and GWAS meta-analyses. Cancer Med..

[22] Sameer A.S., Nissar S. (2021). Toll-Like Receptors (TLRs): Structure, Functions, Signaling, and Role of Their Polymorphisms in Colorectal Cancer Susceptibility. BioMed Res. Int..

[23] CKüper F.-X.B., Neuhofer W. (2012). Toll-like receptor 4 activates NF-κB and MAP kinase pathways to regulate expression of proinflammatory COX-2 in renal medullary collecting duct cells. Am. J. Physiol.-Ren. Physiol..

[24] Zhang X., Yang Y.X., Lu J.J., Hou D.Y., Abudukeyoumu A., Zhang H.W., Li M.Q., Xie F. (2024). Active Heme Metabolism Suppresses Macrophage Phagocytosis via the TLR4/Type I IFN Signaling/CD36 in Uterine Endometrial Cancer. Am. J. Reprod. Immunol..

[25] Gulbay G., Secme M., Mutlu D. (2023). Fusaric acid inhibits cell proliferation and downregulates expressions of toll-like receptors pathway genes in Ishikawa endometrial cancer cells. Eur. Rev. Med. Pharmacol. Sci..

[26] Ashton K.A., Proietto A., Otton G., Symonds I., McEvoy M., Attia J., Scott R.J. (2010). Toll-Like Receptor (TLR) and Nucleosome-binding Oligomerization Domain (NOD) gene polymorphisms and endometrial cancer risk. BMC Cancer.

[27] Allhorn S., Böing C., Koch A.A., Kimmig R., Gashaw I. (2008). TLR3 and TLR4 expression in healthy and diseased human endometrium. Reprod. Biol. Endocrinol..

[28] Hossain M.A., Islam S.M.S., Quinn J.M.W., Huq F., Moni M.A. (2019). Machine learning and bioinformatics models to identify gene expression patterns of ovarian cancer associated with disease progression and mortality. J. Biomed. Inform..

[29] Hao B., Chen Z., Bi B., Yu M., Yao S., Feng Y., Yu Y., Pan L., Di D., Luo G. (2018). Role of TLR4 as a prognostic factor for survival in various cancers: A meta-analysis. Oncotarget.

[30] Lupi L.A., Cucielo M.S., Silveira H.S., Gaiotte L.B., Cesário R.C., Seiva F.R.F., de Almeida Chuffa L.G. (2020). The role of Toll-like receptor 4 signaling pathway in ovarian, cervical, and endometrial cancers. Life Sci..

[31] Yuan Z.-Q., Peng X.-C., Liu L., Yang F.-Y., Qian F. (2025). Olfactory receptors and human diseases. Cell Tissue Res..

[32] Kerslake R., Hall M., Randeva H.S., Spandidos D.A., Chatha K., Kyrou I., Karteris E. (2020). Co-expression of peripheral olfactory receptors with SARS-CoV-2 infection mediators: Potential implications beyond loss of smell as a COVID-19 symptom. Int. J. Mol. Med..

[33] Kerslake R., Hall M., Vagnarelli P., Jeyaneethi J., Randeva H.S., Pados G., Kyrou I., Karteris E. (2021). A pancancer overview of FBN1, asprosin and its cognate receptor OR4M1 with detailed expression profiling in ovarian cancer. Oncol. Lett..

[34] Bailey J., Tyson-Capper A.J., Gilmore K., Robson S.C., Europe-Finner G.N. (2005). Identification of human myometrial target genes of the cAMP pathway: The role of cAMP-response element binding (CREB) and modulator (CREMα and CREMτ2α) proteins. J. Mol. Endocrinol..

[35] Mao L., Tang Y., Deng M.J., Huang C.T., Lan D., Nong W.Z., Li L., Wang Q. (2022). A combined biomarker panel shows improved sensitivity and specificity for detection of ovarian cancer. J. Clin. Lab. Anal..

[36] Pastorekova S., Gillies R.J. (2019). The role of carbonic anhydrase IX in cancer development: Links to hypoxia, acidosis, and beyond. Cancer Metastasis Rev..

[37] Ronca R., Supuran C.T. (2024). Carbonic anhydrase IX: An atypical target for innovative therapies in cancer. Biochim. Biophys. Acta (BBA)—Rev. Cancer.

[38] Xie Q., Huang J., Xie Y., Hu J., Jin L. (2025). Identification of prognostic biomarkers for endometrioid endometrial carcinoma based on the miRNA and mRNA co-expression network regulated by estradiol. Clinics.

[39] Jin M., Yin C., Yang J., Yang X., Wang J., Zhu J., Yuan J. (2024). Identification and validation of calcium extrusion-related genes prognostic signature in colon adenocarcinoma. PeerJ.

[40] Sharma A., Kumar R., Aier I., Semwal R., Tyagi P., Varadwaj P. (2019). Sense of Smell: Structural, Functional, Mechanistic Advancements and Challenges in Human Olfactory Research. Curr. Neuropharmacol..

[41] Boekhoff I., Inglese J., Schleicher S., Koch W.J., Lefkowitz R.J., Breer H. (1994). Olfactory desensitization requires membrane targeting of receptor kinase mediated by beta gamma-subunits of heterotrimeric G. proteins. J. Biol. Chem..

[42] Peppel K., Boekhoff I., McDonald P., Breer H., Caron M.G., Lefkowitz R.J. (1997). G protein-coupled receptor kinase 3 (GRK3) gene disruption leads to loss of odorant receptor desensitization. J. Biol. Chem..

[43] Mashukova A., Spehr M., Hatt H., Neuhaus E.M. (2006). β-Arrestin2-Mediated Internalization of Mammalian Odorant Receptors. J. Neurosci..

[44] Fattahi F., Saeednejad Zanjani L., Habibi Shams Z., Kiani J., Mehrazma M., Najafi M., Madjd Z. (2021). High expression of DNA damage-inducible transcript 4 (DDIT4) is associated with advanced pathological features in the patients with colorectal cancer. Sci. Rep..

[45] Yoshikawa N., Yoshida K., Liu W., Matsukawa T., Hattori S., Yoshihara M., Tamauchi S., Ikeda Y., Yokoi A., Shimizu Y. (2023). The prognostic significance of DDIT4 in endometrial cancer. Cancer Biomark..

[46] Chang B., Liu G., Yang G., Mercado-Uribe I., Huang M., Liu J. (2009). REDD1 is required for RAS-mediated transformation of human ovarian epithelial cells. Cell Cycle.

[47] Ji Y., Xie M., Lan H., Zhang Y., Long Y., Weng H., Li D., Cai W., Zhu H., Niu Y. (2013). PRR11 is a novel gene implicated in cell cycle progression and lung cancer. Int. J. Biochem. Cell Biol..

[48] Ni W., Yi L., Dong X., Cao M., Zheng J., Wei Q., Yuan C. (2023). PRR11 is a prognostic biomarker and correlates with immune infiltrates in bladder urothelial carcinoma. Sci. Rep..

[49] Zhan Y., Wu X., Zheng G., Jin J., Li C., Yu G., Li W. (2020). Proline-rich protein 11 overexpression is associated with a more aggressive phenotype and poor overall survival in ovarian cancer patients. World J. Surg. Oncol..

[50] Dai W., Liu Y., Zhang T., Huang Z., Xu X., Zhao Z., Liu J., Zhai E., Cai S., Chen J. (2023). Spindle function and Wnt pathway inhibition by PBX1 to suppress tumor progression via downregulating DCDC2 in colorectal cancer. Oncogenesis.

[51] Maclean A., Adishesh M., Button L., Richards L., Alnafakh R., Newton E., Drury J., Hapangama D.K. (2022). The effect of pre-analytical variables on downstream application and data analysis of human endometrial biopsies. Hum. Reprod. Open.

[52] Medina-Gutierrez E., Céspedes M.V., Gallardo A., Rioja-Blanco E., Pavon M.A., Asensio-Puig L., Farre L., Alba-Castellon L., Unzueta U., Villaverde A. (2022). Novel Endometrial Cancer Models Using Sensitive Metastasis Tracing for CXCR4-Targeted Therapy in Advanced Disease. Biomedicines.

[53] Ueda H., Ishiguro T., Mori Y., Yamawaki K., Okamoto K., Enomoto T., Yoshihara K. (2024). Glycolysis-mTORC1 crosstalk drives proliferation of patient-derived endometrial cancer spheroid cells with ALDH activity. Cell Death Discov..

[54] Kim M., Lee S., Park W.H., Suh D.H., Kim K., Kim Y.B., No J.H. (2018). Silencing Bmi1 expression suppresses cancer stemness and enhances chemosensitivity in endometrial cancer cells. Biomed. Pharmacother..

[55] Faruqi S.A., Satyaswaroop P.G., LiVolsi V.A., Deger R.B., Noumoff J.S. (2002). Establishment and characterization of a poorly differentiated lethal human endometrial carcinoma cell line (NOU-1) with karyotype 46,XX. Cancer Genet. Cytogenet..

[56] Tang G., Cho M., Wang X. (2022). OncoDB: An interactive online database for analysis of gene expression and viral infection in cancer. Nucleic Acids Res..

[57] Trapnell C., Roberts A., Goff L., Pertea G., Kim D., Kelley D.R., Pimentel H., Salzberg S.L., Rinn J.L., Pachter L. (2012). Differential gene and transcript expression analysis of RNA-seq experiments with TopHat and Cufflinks. Nat. Protoc..

[58] Akhmedov M., Martinelli A., Geiger R., Kwee I. (2020). Omics Playground: A comprehensive self-service platform for visualization, analytics and exploration of Big Omics Data. NAR Genom. Bioinform..

[59] Van Der Maaten L., Courville A., Fergus R., Manning C. (2014). Accelerating t-SNE Using Tree-Based Algorithms. https://jmlr.org/papers/v15/vandermaaten14a.html.

[60] Witten D.M., Tibshirani R., Hastie T. (2009). A penalized matrix decomposition, with applications to sparse principal components and canonical correlation analysis. Biostatistics.

[61] Wang X., Spandidos A., Wang H., Seed B. (2012). PrimerBank: A PCR primer database for quantitative gene expression analysis, 2012 update. Nucleic Acids Res..

